# Reordering the machinery of participation with young people

**DOI:** 10.1111/1467-9566.13426

**Published:** 2022-01-25

**Authors:** Hannah Cowan, Charlotte Kühlbrandt, Hana Riazuddin

**Affiliations:** ^1^ School of Life Course & Population Sciences King's College London London UK; ^2^ Department of Geography King's College London London UK

**Keywords:** co‐production, creative methods, health inequalities, knowledge production, participation, scholar activism

## Abstract

In this article, we reflect on our ongoing work that attempts to redistribute the agenda‐setting powers of researchers, research funders and the complex of private and public partnerships in the biomedical sciences. Despite calls for diversification, the current landscape is dominated by a traditional medical habitus that prioritises discovery science. This has moral and political consequences. Simultaneously, we have seen a slow rise in top–down infrastructures of public participation in medical science. While we are critical of the resulting machinery of participation, we believe in its premise that knowledge and expertise are everywhere. In our research project—called Utopia Now!—we have been seeking to involve young people in deciding the future of biomedical research. However, this project is itself premised on a number of our own complicities with the power held by universities and research infrastructures. Here, we explore three preliminary tactics through which we attempt to make these complicities politically productive, taking into account the limitations of working as early career researchers. We find that our mediation between young people and researchers across disciplines is not only integral for re‐politicising medical research, but also changes our understanding of knowledge production as a process of reordering, sorting and sharing.

## INTRODUCTION

### The participation machinery

It was a Friday in 2019, and a sea of young people were striking from school to fill up the streets of central London. Those participating in the Schools Strike for Climate walked, danced and shouted their way down to Parliament Square with placards: ‘There is no Plan(et) B’, ‘Don't fuck up our future!'. ‘We *are* the future’, they reminded us as we ourselves truanted off work to join the march; young people should have a seat at the table when it comes to deciding how to make the future world a better place. Yet in our various roles in diversity, inclusion and ‘Patient and Public Involvement’ at King's College London medical school, we see few opportunities for young people to have a say in the future. This article discusses our tactics for realigning power in medical knowledge production, and we are especially interested in ways to make space for young people. We agree that they should have input in the construction of emerging worlds, including biomedical ones, given that they are the ones who will be living in the future.

The medical school at King's College London (KCL) is very much embedded in the government's narrative that the UK is well placed to be a global leader in medical and scientific research, given its historic role in the development of biomedical practice (Johnson, 2021). Indeed, KCL prides itself on being one of the oldest medical schools in the UK, dating back to the 12th century. You only have to look to the school's active rivalry in the discovery of DNA to see that the institution's discourse is one of discovery science, in which individual biomedical heroes find some underlying truth about nature that has always been there, but humans are yet to know about. Social theorists, however, have long demonstrated how scientists in fact construct, rather than neutrally ‘discover’ knowledge, creating new stories and artefacts through their work (Mol, 2002; Pinch & Bijker, 1987).

It is, therefore, problematic if those who construct dominant knowledge are only highly educated scientists who have a particular and partial view of the world. From the in‐house pathology museum housing wax models, jars of body parts in formaldehyde and old lecture theatre benches with racist and misogynistic etchings, it is also clear that this medical school has played an active part in formulating the quintessential medical habitus, a mode of being defined by machoism, certainty and a rationalism that excludes emotion (Becker et al., 1961; Hafferty, 1988; Sinclair, 1997). This medical habitus continues to be reproduced (Hafferty & O'Donnell, 2014; Michalec & Hafferty, 2013), resulting in biomedical knowledge that recreates, for example, gender‐, race‐ and disability‐related inequalities by defining which diagnoses and treatments are applicable to which constructed categories of people (Kafer, 2013; Saini, 2019; Shim, 2014). Through scientific attempts to ‘capture’ truths about the body, biomedical knowledge often entraps black, brown, disabled and non‐gender binary conforming people as ‘other’ (McKittrick, 2021). It is clear that university leadership has resolved to change society through research, indeed KCL's strapline is ‘to make the world a better place’ (KCL, n.d.). However, the protection of medical knowledge as something that can only be formed by those with a medical habitus has prevented young people, and especially young black and brown people, from being invited to disrupt what this ‘better place’ is.

There has, however, been a shift: a crisis of legitimacy has been brewing over the past few decades where slowly, quietly, the participation agenda has been gaining momentum in the UK's research institutions. The National Institute for Health Research (NIHR) has been building its argument for Patient and Public Involvement (PPI), based on the compelling definition that PPI should result in research being carried out ‘with’ or ‘by’ members of the public rather than ‘to’, ‘about’ or ‘for’ them (NIHR, 2019). It was initially driven by a democratic narrative that those who could benefit from research should have a say in how it is formulated (Russell et al., 2019). Most medical research infrastructures now mandate PPI, resulting in a bureaucratic PPI‐industrial complex, in which we all find ourselves to be a part, a machinery of participation that includes training courses, advisors and boxes to fill in on funding and ethics forms.

While we welcome the attempt to bring about a more democratic practice in scientific research, its execution has been criticised for its shallow nature (Boaz et al., 2016), its involvement of a narrow demographic (Ocloo & Matthews, 2016) and using participation as a mode of control by co‐opting publics (Cooke & Kathari, 2001). This is in part because arguments for PPI have also leaned towards a more consumerist and instrumentalist narrative, encouraging researchers to consult PPI groups in order to gain strategies for increasing trial recruitment, acquiring ‘diverse’ genetic datasets and maximising uptake of research findings amongst service users (Russell et al., 2019).

It is evidently still a struggle to convince researchers and funders that wider publics should have a say in the future worlds made by medical science. Medical researchers and the machinery of participation continue to work very strictly within the realms of what is financially and logistically possible without researchers and funders giving up too much power. As a result, science is contributing to what Bloch and Adorno (1988) called a ‘shrinking of the utopian consciousness’ (p. 3), where ‘making the world a better place’ has come to be about quick technological fixes which continue to reproduce inequalities and poor relations to the climate. The ‘better places’ that get made continue to be those implicitly constructed by scientists; the machinery of participation is not working. We realise that it is too easy to blame the medical researchers themselves. While change is usually made through a collection of everyday practices, these practices are as ingrained as the old medical habitus itself.

## Reordering the machinery of participation

While we are critical of the participation agenda, we believe in the premise on which it is built, which is that knowledge and expertise are situated everywhere and affects everyone. In our current work, we seek to develop a more substantive and utopian mode of participation which looks to disrupt embedded relations of power, to reopen possibilities and create new realities, as opposed to being trapped in the possibilities of the present (Bell & Pahl, 2018). As Bloch and Adorno (1988) suggest, this means reaching beyond specific technologies to think about the totality of social relations, across spheres of labour and interest. Everything is social, from food banks to data servers (Latour, 2005). This has led us to ask the big, meta‐level questions of medical research: What questions should researchers be asking? What should they care about? What should this ‘better world’ look like? We aim to imagine new, better worlds with young people and open conversations with researchers about the kind of work that needs to be done for research to move in that direction. It is an ambitious project, which is why we called it Utopia Now!

In order to find the most hopeful utopian futures, we were committed to having these conversations with those who most need the world to change. As Bloch and Adorno (1988) explain, hope is ‘the opposite of security… danger is always within it’ (p. 16). We needed to talk to those with better understandings of the insecurities of the present, in order to break out of current possibilities and imagine utopian futures which have the potential to undo unequal relations of power (Levitas, 2013). We decided to start local, by working with young people living in the same space as the university in South London, where widening economic inequalities are clearly visible, and Black Lives Matter marches have targeted the university campus (Salisbury, 2020). Our ambition is to find ways to break the traditional medical habitus, reorder the machinery of participation and move further towards the redistribution of agenda‐setting powers in research. We are glad to see that the university is on board—for example, by funding this project—but doing this work while situated within the machinery of participation has posed challenges.

## Finding our complicities, acknowledging our power

We were confused at first when young people and youth workers referred to us as ‘King's College London’ rather than by our names, (‘just to let you know, King's College London have come to talk to us about a project they're doing, d'you want to join?’). As a small team of early career researchers, we thought of ourselves as small cogs governed by the bigger university wheel. We felt uncomfortable being seen as embodied entities of the very institution we were seeking to critique. But after months of going back and forth between the iron gates of the medical school, which stand proudly and painstakingly refurbished opposite London Bridge Station, and youth centres situated far from transport hubs, wedged under tower blocks, with duff computers, struggling for maintenance costs or fighting to save their space from property developers, we came to admit that of course we are complicit in the material having. We help the university research wheels go round. We benefit from the machinery of participation, and we enable the participation machinery to operate.

We saw our complicities through the lenses of both space and time. Firstly, we had to acknowledge that South London is not just one space; it is many spaces; the same street can mean different things for different people, especially with gentrification on the rise (Riazuddin et al., 2020). With KCL's mission to ‘serve’ the local community, we must be careful not to impose the university's values onto these many spaces, as if they have not yet caught up yet with knowledge, tech, and the better worlds the university tries to impose (Massey, 2005). As Brenman (2021) argues, precarity is not inherent to any particular space. Instead, ‘precarity emerges from bodies and materials assembled in (or out of) place’ (Ibid. p16). With hospitals and universities committing to serve the community on their doorstep, catchment areas can easily become colonised, whether it is by the university's ambition to transform young people into young scientists (with the accompanying medical habitus), or by promoting the diversity of the neighbourhood to pharmaceutical companies for the purpose of collecting biological samples (Guy's & St Thomas’ Biomedical Research Centre, n.d.). Instead, we have tried to understand how young people have developed their own ways of knowing, being and doing and have constructed their own various spaces.

Secondly, we found that the temporal order of research infrastructures is not always well aligned with the timeframes of the people we work with (Sharma, 2014). Even as we start writing this research paper, we know it is planned to be published in two years’ time. Many of the young people we have been working with over the last few months need change to happen more immediately and may have completely different concerns by the time this article is published. While our writing is of course still important for those immersed in an academic temporal order, we must take stock of the often hidden temporal misalignments in which we partake, and think about whose time is serving who. These different makings and occupations of space and time result in different interests and different priorities, to which we have tried to listen and adapt to. But while we are complicit in the spatial and temporal politics of wealth disparity and knowledge production, we are also early career researchers on short‐term contracts with our own intersections of disprivilege. When we attempt to redistribute the university's wealth in joint grant applications or projects, or speed up bureaucratic processes, we are rarely at liberty to do so.

In this article, we discuss our tactics for making our complicities politically productive while remaining situated in this somewhat power‐limited position. For us, making complicities productive means finding ways to redistribute power between the vastly differing spaces and temporal orders we have described. This work, we emphasise, is very much in process, and so as well as assessing what our work to date means for knowledge production, we also Illustrate where it has led us to re‐evaluate our research practice.

## OUR APPROACH

So then—methodologically speaking, how do we seek to redistribute agenda‐setting powers? We recognised that we needed a utopian methodology that challenges modes of knowledge production in the university, which encourages us to dare to imagine new worlds. We did this by drawing on Levitas (2013) ‘utopia as method’, which first means excavating the issues (how do inequalities get done?) before imagining what a better world should look like, to ensure these new imaginaries break with the materialities of current political and research landscapes. We then drew on participatory action research, which has stronger roots in both the arts and Latin America and focuses on redistributions of power within the research process (Cornwall & Jewkes, 1995; Freire, 1970; McQuaid & Plastow, 2017). Specifically, we decided to use creative participatory methods—including drama, film, computer games and fiction writing—because encouraging people to exercise their imagination in itself is important for developing transformative conversations and action (Mcquaid & Plastow, 2017).

Using utopia as method is not about creating a blueprint but using utopia as process, as an exercise to explore potential new ways of being. It is a hopeful practice, grounded in Bloch's (1986) imperative that we as scholars have a responsibility not just to critique the world, but to collectively think with hope to create a better one. But, we also acknowledge that we cannot battle political melancholia without political desire or action (Emcke, 2019). As many activist scholars have pointed out, hope is worthless without some movement, some action (Cornish, 2021; Freire, 1970; Ruszczyk & Bhandari, 2020). We, therefore, decided to work ‘within, against and beyond’ the university (Bell & Pahl, 2018), meaning we must reflect and act on our practice within the research, rather than placing ‘impact’, as is traditional, in the afterwards of the research through dissemination. This is why we have also tried to use a more accessible writing style in this article itself—to transform academic spaces from within. We want to imagine and then work out how to get to better worlds.

The ethics of carrying out action research which seeks to redistribute power are of course more complex than the bureaucratic steps we had to take for university ethical approval (given by King's College London). As part of our methodological practice, we have, therefore, built on Pols (2014) work to create an ethics of care. We try to work with young people on their own terms. We pay attention to the differing normative expectations of all involved to develop ‘a way a living together’ (p81). Here, ethical practice is localised to particular situations and relations between researchers, young people, youth workers and parents; it is about taking on Mol et al. (2010) practice of ‘tinkering’, and looking out for and acting iteratively with young people's needs and preferences, whether this is working around their timetables, providing lunch during workshops or listening to their advice on facilitation.

Our primary methods are participant observation and analysis of creative outputs of a range of arts‐based workshops with young people in which we encouraged them to explore their hopes and fears for the future. This included a 5‐day drama workshop held in February 2020 at Theatre Peckham, a community youth theatre in South London, where eleven 10‐ to 14‐year olds wrote, rehearsed and performed plays about the year 2070. This workshop is the main focus in this article. We also held a series of film‐making workshops, a science fiction story competition and workshop, and a series of Minecraft workshops, all held between Autumn 2020–Autumn 2021 with 7‐ to 16‐year olds, which have also informed our work. Some this work was carried out prior to the COVID‐19 pandemic, some was postponed to do it in person, and some was adapted to take place online.

### Tactic 1: Using health to talk about inequalities

In preparation for the Utopia Now! funding application, we tested out our ideas with a group of 14‐ to 16‐year olds who were attending a biomedical summer school. As part of their week‐long programme, we asked them to think about a utopian future for 2070 relating to health and technology and then present these scenarios back to the group on the final day. We emphasised, along with all good scholars working in the sociology of health and illness, that the things in our society that affect our health are broad and could include education, inequalities and housing.

The teams did some last‐minute shuffling in urgent whispers as some of their parents entered the room to watch their final presentations. One by one, Team Genetics, Team AI, DNAwesome and Team Lifechangers came on stage to present their hopes and dreams for the future. We saw a fantastic play in which a real and a robot doctor had a face‐off in a future daytime chat show. Mainly, though, young people presented their ideal gadget inventions for the future. There was a picture of a flying ambulance, with a rocket launcher on the back, to ensure it could lift off when it got stuck in traffic. We saw surgical equipment that could operate from outside the body. One of the teams presented a diagnostic mirror: one look in the morning would tell you all your diagnostics and automatically book you into any medical appointments it thought you needed. This mirror, they told us, would be expensive. Only the rich could afford having one of their own. But don't worry, people in council housing would still have access: the diagnostic mirror would be located in the corridor, shared by the residents.

The diagnostic mirror in the council housing corridor stuck with us. Our explicit aim had been for young people to come up with their own utopia that would not be bounded by the constraints of our current economic system. We had asked them to think about health and had even emphasised that inequalities may not exist in this utopia, yet, just as with scientific endeavours, they had narrowed utopia down to imaginative, fantastical, contraptions and techno‐fixes. They had not changed the future social relations in which these inventions would operate, and felt unable to abolish economically stratified housing, to rethink social and economic inequalities. It made us think of Mark Fisher’s (2009) dictum, that it is easier to imagine the end of the world, than the end of capitalism. After this pilot exercise, we sat down to think about how we could get young people to imagine the future of technology more broadly and how to approach health from a more oblique angle, in order to help them think outside of current socio‐economic frameworks.

As part of this reflection, we began to critique our own focus on health. One of the reasons we have positioned ourselves within a medical school is because this is where it is possible do research about inequalities, because this is where the funding is. The societal importance attributed to health research can be seen by the sheer difference in budget and breadth of funders available between health research and non‐health‐aligned sociological research (Cockerham & Scambler, 2010, pp. 3–5). Among academics, it is well known that projects have a better chance of funding when they link their project to health inequalities (ideally when these can be made measurable, through comparable mortality or morbidity outcomes) than other kinds of inequalities, such as housing, education and economic disparities; that is, until you link the effects of these inequalities back to health. Since the Black Report (Department of Health & Social Security, 1980), the Whitehead Report (1987) and the Marmot Report (2010) in the UK, inequalities seem to matter more when they affect people's health—and by extension, the costs they create for the health system, the opportunity costs to the economy (Wilkinson & Pickett, 2009). Linking inequalities to health makes them politically palatable, perhaps in part because of the seemingly objective outcomes, the money involved in the medical‐industrial complex, and because of the UK's embedded history of being a welfare state.

While we do believe that it is important to recognise and criticise when inequalities come to affect health, we *also* see it as a political tactic to convince those in power that social and economic inequalities are immoral and problematic; they come to matter. In addition, we are certainly not alone amongst sociologists of health and illness in recognising this; it is one of the ways in which sociology of health and illness lends itself to activist scholarship. It is for this reason that we decided to de‐emphasise health, allowing young people to address wider issues that concerned them personally. It would be our job as medical sociologists to link their concerns back to social determinants of health. Our tactic is to translate the research aims between research participants, funders, policy makers and scientists and understanding when it is useful to operate under the more apolitical mantel of health research, or when we need to talk more explicitly about inequalities and the politics of distribution. It is through this tactic that we feel we can make our project both politically useful, while also being fundable, visible and potentially impactful.

So, when we advertised our Utopia Now! drama workshop, it was for young people to write a play that was simply about *the future*, not the future of health or medicine, or even wellbeing. With the help of our partners, Theatre Peckham, we got a group of young people to sign up for a week of classes. The space intrigued us. It felt like a fairly swish and professional theatre, after walking through a classic South London housing estate. It turned out to be the social responsibility part of the student accommodation block above, built by a London property developer, who were no longer responsible for the front doors that kept breaking amongst other maintenance costs the theatre struggle to afford. In the studio theatre we would hired, the group learnt to come up with ideas, write, rehearse and perform a play. We wanted them to roam free with their ideas and explore their utopias and their dystopias without imposing the constraints of our research interests. When we put this tactic to Mathijs, the actor we were collaborating with to facilitate the workshop, he told us to hold off asking the young people about the message of their plays. ‘That shouldn't be the starting point, we want them to think creatively and only afterwards think about meaning or else we risk the message becoming forced or contrived'. We took on Mathijs’ advice. One group imagined a world in which people communicated via telepathic phone calls and teleportation; and another in which there had been an invasion of robots who had been created by humans and subsequently quarantined on Mars. After the end of the second day we debriefed, wondering what their visions said about health and wellbeing. But then, we turned to the ‘story maps’ that young people had been working on and saw that one of the young people had written: ‘telepathic communication—never lonely’, twice underlined.

As the plays developed, it turned out the robot group were cautioning against the overreliance on robot labour, especially in caring roles, and another group developed a play called ‘Air for Sale’, imagining a polluted future London in which poor people could not afford clean air that was now only available in tanks. These plays were not only inventing telepathic communication, robots and air canisters, they were also consciously critiquing a totality of social relations. In addition, indeed we often thought back to the play on communication and loneliness during the ensuing pandemic, wondering who did and did not have access to the kind of technology that would keep them connected to friends in the long period of social distancing and isolation.

What we found, then, was that when we asked young people directly about the future of health and technology, their imagination moved towards gadgets without thinking about the social context. After the pilot we decided to give young people's imagination more space, rather than limiting the task to thinking explicitly about health, and the result paid off: the plays gave a much more nuanced and (as it turned out) foreboding vision of the future, in which the life of gadgets was enmeshed in a socio‐economic context. In framing our projects to the funders as being about health, we of course make ourselves complicit in a narrative/discourse, in which inequalities only matter when they affect health. We see it as our task to make this complicity productive by bringing these expressions of inequality back into the centre of medical knowledge production.

### Tactic 2: Re‐aligning temporal orders, research can be fun

‘But this project can't just be about having fun activities for kids—where is the research in what you're proposing?’ We were on a call with a grants manager, negotiating which costs in our project could or could not be justified. We ran to the defence, detailing all the data we would be getting and how each method would help us answer different parts of our research question.

Minecraft, drama, fiction, documentary film‐making… even for qualitative researchers these modes of working would not usually be labelled as ‘methods'. They might be used for disseminating findings or as engagement practices, but for data collection? Where are the focus groups, the interviews? Not even video diaries? Our methods are not usual ethnographic practice either. We are not merely observing life as passive researchers, or even active researchers who inevitably affect what is going on. We are asking young people to stage a part of their imagination, in order for us to observe it, in order to make it visible for others. We could see our practices align with researchers working in the arts (Bell & Pahl, 2017; McQuaid & Plastow, 2017), but our methods were seen as out of place in the medical school.

We originally chose these methods because we wanted young people to be able to exercise their imagination. Only later did it occur to us that using these kinds of creative methods are also a way of doing action research. We soon found that community centres were not interested in the Utopia Now! project so much because of its long‐term aims, although they found these interesting. They were engaged because they saw the resources and infrastructure to put on some exciting workshops for their young people, perhaps with the opportunity to interact with academics at the local university. Indeed, it soon became clear that the slow work of medical sociologists, trying to shift agendas and working towards the redistribution of power, was misaligned with the priorities of those working more directly with young people: our temporal orders were askew (Sharma, 2014).

During the drama workshop we experienced how, with an ethics of care, we were able to tinker (Mol et al., 2010) and work out a way for our temporal orders to sync. Mathijs made it clear to us that the research was not the main priority: ‘My primary goal is that the kids have fun. If they go away from this drama workshop having had a nice time, then I’m happy,’ he said with a twinkle in his eye, knowing that he was provoking our academic intensions. ‘Then I hope they learn something, about acting, about writing stories, about the ethics of science. And then last, I hope you get what you need for your research'.

The first surprise during the workshop was that everyone who had signed up actually showed up. The space felt good, everyone started to ease in with warm‐up drama games, and the young people shared their thoughts on classic sci‐fi movie clips and played with creative story‐making. But even so, by the end of the first day we were nervous: Would this study return the richness of research findings the University was expecting?

As we were setting up the second day, hoping more than anything that all the young people would come back, we asked Mathijs to squeeze a twenty‐minute discussion into the schedule, to ask all the participants more directly about our research question: what were their personal hopes and fears for the future? This gave us enough material to feel like at least we had *something* to show for the money. In the end, the mind mapping session turned out to be fun (one of the young people even said this was their favourite part of the workshop), but also an exercise that led nicely into devising their own plays. As the week went on, and as the young people's plays developed, we saw how much more there was to this creative way of working and how much more data we got from it than what we could glean from the interjected focus group discussion. We realised that with some care and by tinkering with the schedule as we went along, we managed to ensure the young people enjoyed themselves both in the here and now and that they also had opportunity to express their thoughts and ideas about the world for the longer‐term aims of the research project.

Friday was the final day of the workshop and also the day of the final performance. At this point, our concerns, too, became very much situated in the present. We were elated that none of the young people had dropped out during the week. One of the participants’ mothers told us how much she had loved seeing her son coming out of his shell, being as outgoing in public as she knew him to be at home. After the final performance, as soon as everyone had left, we rushed to look at the feedback forms. All of the young people said they had learnt something. They said they had felt welcomed, comfortable, and one of them even said they thought we were ‘cool'. Of course, this was mostly Mathijs’ doing, but we felt proud as a team and proud for having chosen and worked with an actor who set the tone just right.

Part of finding ‘ways of living together’ (Pols, 2014, p. 81), of aligning our temporal orders, was to acknowledge the resource gap between the university and the youth centres we were working with. We ensured there was money for the community centres for their time and space, vouchers for the young people to come back for another session at the theatre, lunch as we were in a high food‐poverty area and the option of named acknowledgement. It also meant thinking together with community youth organisations, asking young people about when they might be available and planning around school timetables and holidays.

But realigning research and play was also integral for ensuring our timelines were in sync. It turned out that research and fun, work and leisure, do not have to be separated into different spheres; capitalist means of production have not only divided labour between people (Marx, 2000, p. 78), but divided our monotonous work from our leisure time, as if one is *in service to* the other. Rather, fun, and creating safe space to play, is something that should be incorporated into the very activity of research. These methods do of course require a lot more labour than setting up a focus group. We had to keep up, be adaptive, and tinker. This is one way in which we have been able to make our dual complicities productive, both working in a resource‐rich research infrastructure, and being situated in a long‐term temporal order of inquiry and change.

### Tactic 3: Mediating space

Our third tactic was more explicitly formed in the planning stages of our research project. We were aware that space and geopolitics were going to be important facets to distributing power. We did not only want to bring young people's voices into centres of power at the university, which perhaps aligns with more traditional notions of participation. We wanted to also disperse the centre of power and force those seated here in the medical school to leave and go to other places where young people spend their time so that researchers could understand the worlds which they are ultimately co‐constructing through their work. We had, therefore, planned to evaluate the power dynamics brought about by different spaces. Our assumption here was that young people might be more intimidated by the polished setting at KCL, but more comfortable in a space they had chosen for themselves. We wanted to play with the power dynamics of different spaces, to see where people felt comfortable and where they were able to vocalise their thoughts. Once we hit pandemic times, we had to completely rethink how we brought researchers and young people together. But even before then, we found that getting everyone in the same room was hard and starting a dialogue even harder.

Our initial realisation that this was going to be more difficult than we thought came on the evening of the final performance for the theatre workshop, our first attempt to bring young people and researchers other than ourselves together. The Friday afternoon was full of nerves, as the young people finally rehearsed their completed plays on stage during the tech‐rehearsal, working out the light and music cues. We were equally nervous, not just for them, but because we started to worry that none of the researchers we had invited to the theatre would show up: would we be able to fill out the room? would the young people feel like they had an audience? We had put the invitation for researchers wide out there, but we had also carefully selected some researchers to come who might be particularly interested in the plays; someone from the human brain project for the robot group, an ethnographer of pollution for Air for Sale, a transport sociologist and an urban planner for the teleportation group. At 4:30, half an hour before the start of the show, we started to get texts and emails from friends and colleagues to say they were not going to show up—they were ill, too busy, or just were not going to be able to make it after all. This was not a personal failing on their part. Instead, it illustrates the polarity of the worlds we are trying to have meet. The location of our event mattered: the youth theatre was a good thirty‐minute journey away from the university; it was not like they could just pop down from the office for an hour after work. With workloads heavier than contracted hours, and this kind of activity being seen within universities as ‘extra‐curricular’ rather than a vital part of the work itself, it is perhaps unsurprising these researchers and young people rarely share a space.

We had chosen to do the final performance in the smaller studio theatre, and so to our relief, once all the parents got there, the room felt full, and we could rest assured that the young people would have an audience for their final performance. Five researchers also turned up. We were halfway there: academics and young people at least in the same room together. But the next hurdle, as we soon found out, was facilitating the conversation. After the three performances, all the young people came up to the front to answer questions about their plays. We had worked with them the day before to think critically about the stories they had created, and had pre‐emptively thought about how they might discuss some of the questions that might come up. We opened up the floor to questions to that slow awkward silence when no one wants to go first. Aha! We had anticipated this; we had already prompted one of our colleagues with a question we knew one of the groups was prepared to answer. But after this first question the silence returned. Another question from one of the researchers, thank god for their participation! It was an engaged but quite complex one that Mathijs rephrased before the young people answered and then another from a medical scientist, and there was even a bit of back and forth between them and the young people with some facilitation. But then, something we did not expect: ‘you do know teleportation—well, it's not physically possible?’ We realised that, as social scientists, we had not prepared the young actors for a scientific question. It was great to see researchers taking young people's work seriously, but we had not realised the enormity of the translation work for such a multi‐faceted conversation to flow. ‘Yeah'… they hesitated, ‘but it's a utopian world, it's not real’. We interjected here—'but tell us about what the void you got stuck in represents—yesterday you told us the void was kinda like a metaphor?’ One of the young people smiled and told the audience that the void was a bit like when your phone runs out of battery and you feel totally disconnected, and the whole play is really about a world where people are less lonely and more connected, another contributed.

It was only afterwards that we realised that we had spent time and energy preparing the young people to engage with the researchers, anticipating particular questions and building their confidence and that in the process we had not thought to put in the same effort to prepare ourselves for interdisciplinary conversations or the researchers to engage with young people. We also realised that none of the parents asked any questions. We tried to encourage them, another moment of silence, but no one came forward. There was no free‐flowing conversation. It seemed that although we had flipped some of the power relations, where it was the young people who were the ones answering rather than asking the questions, there was still a disconnect between medical researchers, social scientists, parents and young people that needed bridging, that required translation from us and Mathijs. While we were all sharing the same space, the divide between academics and young people was, if anything, more apparent.

As we continued our work online over lockdown, we found again and again that space matters, but it is also important for us, the artists, and the youth workers we work with, to be able to mediate and translate within that space. In preparation for our zoom workshops, young people told us that in order for them to feel comfortable talking online, it would be better for us to ask each person in turn to speak, instead of asking a question and leaving someone to respond first. They were happy to contribute, but they wanted us to moderate and chair the conversation.

The more online workshops and events we did with young people, the more apparent this became. Zoom is not a non‐space. Like South London, it is many spaces. It is people's bedrooms, kitchens and homes. It is home offices for those who have one and electronic backgrounds for those who can find a neutral wall in their home and have a computer fast enough to generate it. For many of the young people, Zoom was siblings saying hi, learning instruments or screaming in the background, mums interrupting. Some kept their cameras and/or mics off, or carefully curated the space, to conceal their now not‐so private home lives. For others, online meetings are inaccessible spaces: many young people could not join our workshops because they did not have access to technology; and even when they did, they did not necessarily have internet that allowed for a smooth ride. Zoom reveals who has the good tech and who does not. With private lives becoming so openly legible, we found that it was even more important to make the virtual room a safe and welcoming space, to mediate between all the different places that were lined up on our screens. We are still working out how to do this, but offering people the opportunity to speak in turn, with the option to pass, is one of these. Adapting to when people want their cameras or microphones on or off is another, and learning new online‐specific social cues for when people are on the edge of wanting to say something. We have also learnt from some of our artistic collaborators who have successfully reordered power dynamics by orchestrating who gets to speak, participate and when.

Those working in the PPI‐industrial complex have long acknowledged that it is important, but also hard, to get people from different positionalities in the same room, whether physical or virtual. There has been less discussion about how the kind of space people gather in really matters. The young people we spoke to told us that in order to develop more meaningful relationships with researchers, they would prefer initial meetings to be in their own space, their community centre, because it is a place they feel safe and at ease. Though youth centres are threatened by property developers and high maintenance costs, they are often safer places for young people than King's College London; safety is not (or not only) about a temperature‐regulated office with working IT equipment. Just as on Zoom, no physical space is in fact empty, and no space is in and of itself safe. Space is made up of the things that happen in it. Bringing researchers into young people's spaces changes it and reintroduces an element of precarity with stilted social relations. In this instance, we found it is the mediators, the translators, who can make spaces more or less safe.

Structural inequalities cannot simply be broken down through skilled chairing, but it is possible to talk across differences and ensure the ways of being and knowing which are produced in spaces other than the university are heard. Given the underlying power dynamics, we are ultimately trying to break down, ensuring that researchers really listen to young people and that young people really feel able to speak out is a difficult business. We cannot blame researchers for not having these skills; they are not taught them because they are not seen as valuable. We are still in the process of learning these mediation skills ourselves. Many artists and youth workers we work with have developed them already and we can learn from them. It is all part of a longer process; reordering the participatory machinery in an old medical school is slow work.

## DISCUSSION

In this article, we have discussed how we have tried to use our positions within the participation machinery of a medical school to challenge and begin to reorder the machinery of participation in UK‐based biomedical research. Whilst we are early career researchers in somewhat power‐limited positions, we illustrated three tactics which we developed to moderate the continuities between health and inequalities, different timelines and different spaces. These tactics required us to be mediators between young people, natural scientists and social scientists. In going back and forth, looping in different people's ideas and going back round again, we have started to ask ourselves: what are we as researchers really adding through our mediation? What new, original knowledge are we creating? In this constant shifting, toing and froing, we have come to realise that perhaps knowledge production is really just this: the reordering, sorting and sharing of knowledge across different spaces, different temporal orders and different ontological, epistemic and moral regimes.

Yes, as so many medical sociologists have reiterated, knowledge is not discovered; it is not already sitting there fully formed, waiting to be captured. But at the same time, knowledge is not produced and constructed out of nothing; relativity is not about materialising things out of thin air. The raw materials, as infinitely divisible parts, are already there, assembled in some way or other. Knowledge production is then about how these materials are re‐assembled and reordered. We can be a lot more imaginative with how the pieces are put together, how the edifice is carved up and how we think about who is served by what kind of knowledge. But as young climate change activists have reminded us, we have to work with the world, the resources, we've got; there is no plan(et) B (Thunberg, 2020). We must attend to the relations between humans, multi‐species worlds and material things (Livingston, 2019; Tsing, 2015).

The young people we have worked with know a hell of a lot about these uneven relations both between people and between humans and the climate. They are wise. But, their knowledge about inequalities and how they come about is not in the right places, often is not part of the same timelines, and (although it does happen), it is not always mediated by theory, history and context. Equally, the biomedical researchers we work with know a hell of a lot. They are wise. In addition, while there is a certain amount of enthusiasm for a different kind of knowledge production, the things that scientists know are often not mediated by the knowledge of the world they are working with; how their research translates into the everyday lives of people. In our work, we are trying to pay particular attention to the ways in which the world gets constructed, how that construction happens and to intervene to ensure that social inequalities are taken into account in how we construct better worlds.

To be clear, the tactics we present in this article are still very much in progress, and they are just a start. But, we hope to have drawn closer attention to how, where and when this construction takes place, and who gets to take part. We are just beginning to put together these tactics, but what we do know is that learning how to be good mediators is a key part of what academics need to do to take participation seriously. We need to share work across more interdisciplinary and creative practices, not to capture knowledge but to unsettle old ways of thinking and create some ever‐morphing new (McKittrick, 2021). Working between different spaces, timelines and understandings of the world should not only be an extra‐curricular activity for medical scientists and academics in general; it should be an integral part of research practice that is taught in basic research training. Participatory practices also need to take a step back, to think about the worlds we should be creating rather than simply making small adjustments to existing directions of research. In order to do this well, it is imperative that research institutions work with others to understand the things that need to be deconstructed in the world, in order for us to be able to develop a genuinely better one for those that really most need the world to change. In doing so, we must acknowledge that the world is both many and one. We must take the realities made in different spaces and logics seriously, ensure we do not colonise them with university logics, but also acknowledge that none of these spaces are siloed—our actions have impacts across boundaries. In other words, medical schools have got to be more ready to change their own internalised biomedical logics, to consciously challenge their own assumptions, in order to redistribute power, and collectively build new and emerging worlds.

## AUTHOR CONTRIBUTIONS

**Hannah Cowan:** Conceptualization (equal); Data curation (equal); Formal analysis (equal); Funding acquisition (equal); Investigation (equal); Methodology (equal); Project administration (equal); Writing – original draft (lead); Writing – review & editing (lead). **Charlotte Kuhlbrandt:** Conceptualization (equal); Data curation (equal); Formal analysis (equal); Funding acquisition (equal); Investigation (equal); Methodology (equal); Project administration (equal); Writing – original draft (lead); Writing – review & editing (lead). **Hana Riazuddin:** Conceptualization (equal); Data curation (equal); Formal analysis (equal); Investigation (equal); Methodology (equal); Project administration (equal); Writing – review & editing (supporting).

## Data Availability

Research data are not shared due to ethical reasons.
